# Editorial

**Published:** 1838-01-03

**Authors:** 


THE
### MEDICAL EXAMINER.
PHILADELPHIA.

Wednesday, January 3, 1838.

Conformably to proposals some time since put forth, the first number of the Medical Examiner is to-day issued. Its objects were announced to be, to place in the hands of the profession a cheap and succinct compendium of valuable matter, necessarily not embraced in the periodical works, published at greater intervals of time. For the accomplishment of this object, the editors believe, that they have before them a rich field of investigation, the cultivation of which has been hitherto neglected. It is their intention to devote a large space of their columns to reports of clinical lectures, delivered in the hospitals of this city. This plan has been elsewhere pursued with signal success. It has had the effect of rescuing from oblivion instructive lessons of experience, of stimulating the lecturer to renewed exertion; and, in many instances, of banishing indolence and incompetency from the wards and amphitheatre. The profession at large is deeply interested in effecting these results. To the student of medicine, and to the practitioner residing out of the reach of hospitals, a regular and accurate report of the clinical practice and lectures of the American metropolis of medicine, must be of special importance.

It will be the aim of the editors to render the Examiner a general receptacle of interesting matter, in all the departments of medicine and surgery, and a faithful chronicle of passing events, worthy the notice of the profession. A temperate, but independent discussion of questions involving medical politics will be maintained. The columns of this journal are open to the communications of all parties, and to the expression of every shade of opinion.

In concluding this brief outline of the objects which we hope to accomplish by the establishment of a new medical journal, we may be permitted to express our steadfast determination to devote our humble labours and undivided energies to the promotion of the best interests of the *entire* profession, to which we have the honour to belong. These interests we will advocate under every circumstance, and at any sacrifice.

---

It is our painful duty to commence our editorial career with the badge of mourning. Dr. PHILIP SYNG PHYSICK departed this life at twenty-five minutes past eight o'clock, on the morning of Friday, December 15th, in the seventieth year of his age. This great and good man has passed away, full of years, and full of honors, amid the respect and regret of an entire community. His death will, we believe, be looked upon as a national loss by the whole American public. In this city all proper homage has been paid to his memory. The faculties of medicine of the University of Pennsylvania, and of the Jefferson College, suspended their duties. The students of the former institution have requested Professor Chapman to deliver before them an eulogium on the deceased. Dr. Chapman has signified his intention of complying with this request, during the course of the present session. Professor Horner has been selected by the American Philosophical Society, of which Dr. Physick was a member, to prepare an obituary notice of his late colleague. The Philadelphia Medical Society, of which Dr. Physick was, at the time of his death, the venerated President, have named Dr. Jacob Randolph, of this city, to fulfil a similar duty.

With these promises before us, we postpone a notice of the life and professional career of Dr. Physick. In reviewing hereafter the labours of the gentlemen to whom this task has been assigned, we shall have occasion to lay before our readers full details upon this interesting subject.

---

It is our wish to impress the stamp of catholicity upon the matter of our journal. We therefore invite the communications of our professional brethren from all sections of the union. From those whose active duties are incompatible with the preparation of elaborate essays, we ask the contribution of short summaries of cases and observations, which they may deem interesting. For publications of this nature, the columns of a medical gazette are particularly adapted. We have secured a trans-atlantic correspondence which will enrich future numbers.

---

We commence to-day the publication of a series of Professor Chapman's lectures, reported for this journal, by the permission and under the supervision of the author. We propose shortly to lay before our readers a course of lectures on Midwifery, by Dr. C. D. Meigs, of this city. This popular course Dr. Meigs has kindly offered to prepare himself for our columns.

---

We shall be pleased to exchange with any journal disposed to reciprocate.

---

Gentlemen, who receive gratuitously the first number of the Examiner, will confer a favor by circulating it. Those, who are disposed to become subscribers, are requested to forward a remittance, according to the terms of our advertisement, in the manner therein expressed.

